# miR-21-5p Inhibits Ferroptosis in Hepatocellular Carcinoma Cells by Regulating the AKT/mTOR Signaling Pathway through MELK

**DOI:** 10.1155/2023/8929525

**Published:** 2023-03-24

**Authors:** Zongqiang Hu, Laibang Li, Ma Li, Xibing Zhang, Yu Zhang, Jianghua Ran, Li Li

**Affiliations:** ^1^First People's Hospital of Kunming City, Kunming 650032, Yunnan, China; ^2^The Calmette Affiliated Hospital of Kunming Medical University, Kunming 650032, Yunnan, China

## Abstract

**Background:**

Hepatocellular carcinoma (HCC) is one of the most prevalent cancers, and its incidence rate is increasing worldwide. At present, there is no ideal treatment for HCC. In recent years, molecular-targeted therapy has shown significant therapeutic benefits for patients. Ferroptosis is a modality of regulated cell death, and previous studies have found that inducing ferroptosis in liver cancer cells can inhibit the progression of liver cancer. The aim of this study is to investigate the regulatory mechanism of miR-21-5p in regulating ferroptosis in HCC cells.

**Methods:**

CCK-8 was used to measure cell viability, EdU and colony formation were used to measure cell proliferation, and Transwell assays were used to measure cell migration and invasion. RT-qPCR was used to detect the level of miR-21-5p, Western blotting was used to detect the protein expression level, a dual-luciferase reporter gene assay was used to determine the targeting relationship between miR-21-5p and MELK, and coimmunoprecipitation was used to determine the interaction between MELK and AKT.

**Results:**

Overexpression of miR-21-5p and MELK facilitated the viability, proliferation, colony formation, invasion, and migration of HCC cells. Downregulation of miR-21-5p suppressed the level of MELK and the progression of HCC. MELK regulated the AKT/mTOR signaling pathway, causing changes in the levels of GPX4, GSH, FTH1, *x*CT, heme oxygenase 1(HO-1), reactive oxygen species, and Fe^2+^ to regulate the ferroptosis of hepatoma cells. Erastin, an inducer of ferroptosis, attenuated the repressive influence of miR-21-5p on ferroptosis in HCC cells.

**Conclusion:**

In summary, this study demonstrates that miR-21-5p inhibits the ferroptosis of HCC cells by regulating the AKT/mTOR signaling pathway through MELK.

## 1. Introduction

Hepatocellular carcinoma (HCC) is one of the most prevalent cancers, and its morbidity is increasing worldwide. At present, there is no ideal treatment for HCC, and the prognosis of HCC patients is very poor. Many researchers are trying to improve the treatment effect by changing the treatment strategy [1]. Ferroptosis is a modality of regulated cell death characterized by an iron-dependent accumulation of lipid hydrogen peroxide to cell lethal levels. Since most of the body's excess iron is stored in the liver, the liver is crucial in maintaining iron homeostasis. Proteins that control the cell cycle have been reported to be regulated by iron levels [2]. Therefore, cancer cells show an increased demand for iron to promote their proliferation, and this iron dependence makes cancer cells more susceptible to ferroptosis, leading to cancer cell death [3–5]. In addition, the tumor inhibitor p53 is a transcription factor that is activated in response to cellular stress, and its expression is also regulated by altered iron metabolism [6]. Therefore, the induction of ferroptosis in HCC cells can inhibit the progression of HCC, and exploring the regulatory mechanism of ferroptosis in HCC cells may promote the evolution of targeted interventions for HCC. Inducing ferroptosis may be a prospective approach for HCC therapy.

MicroRNAs (miRNAs) are small (∼20–24 bp), highly conserved noncoding RNAs that are expressed endogenously in each cell type and bind to target mRNAs to participate in the posttranscriptional regulation of gene expression [7]. It has been shown that miRNAs participate in HCC initiation and progression as oncogenes or tumor suppressor genes, and specific miRNA overexpression and interference can be used to study the influence of miRNAs in cancer initiation and progression [8]. miR-21-5p has been shown to be involved in various pathological processes, including proliferation, mesenchymal transition, metastasis, and apoptosis [9], and acts as a regulator in a variety of diseases, including colorectal cancer [10], breast cancer [11], thyroid cancer [12], myocardial injury [13], and acute kidney injury [14]. In addition, various miRNAs have been found to be closely related to ferroptosis, such as miR-17-92, which protects endothelial cells from ferroptosis [15]. miR-522 inhibits ferroptosis in gastric cancer cells [16]. miR-137 regulates ferroptosis in melanoma cells [17]. At present, there are few reports on miRNAs regulating ferroptosis in HCC cells. Cho et al. [18] found that miR-21-5p was overexpressed in HCC and associated with the prognosis of HCC. Therefore, it is possible that miR-21-5p may regulate the ferroptosis of hepatoma cells, and the study of miR-21-5p may aid in understanding the mechanism of ferroptosis of hepatoma cells.

Maternal–fetal leucine zipper kinase (MELK) is involved in many cancer-related processes, including chemoresistance, stem cell turnover, and tumor growth [19]. Many studies have demonstrated that MELK is a cell cycle regulator that is critical for mitotic progression [20]. MELK is overexpressed in various cancers, and high MELK expression is associated with infaust prognosis and has been identified as a prospective target for multiple cancer types. Guo and Zhu [21] found that upregulation of MELK was associated with the prognosis of HCC. Xia et al. [22] showed that the overexpression of MELK was nearly interrelated with the early recurrence of HCC and the survival rate of patients. Yang et al. [23] used comprehensive bioinformatics analysis to demonstrate that MELK may be an underlying target for HCC. Moreover, some studies have indicated that miRNA is involved in the regulation of the expression of MELK in HCC. Li et al. [24] found that miR-214-3p can regulate its target gene MELK by binding immediately to MELK-3′-UTR, and downregulation of MELK expression can inhibit the progression of HCC. In a recent study, the pharmacological inhibition of MELK was found to limit ferroptosis and inflammatory responses in colitis and colitis-triggered carcinogenesis [25]. Therefore, it is possible that MELK may participate in the regulation of ferroptosis in HCC cells, and this regulation may be regulated by miRNA.

The protein kinase B/mammalian target of rapamycin (AKT/mTOR) pathway plays a vital role in the evolution of HCC in various biological processes, incorporating proliferation, metastasis, chemical and radiation resistance, energy metabolism, and autophagy [26]. PIP3, as a second messenger, facilitates the activation of AKT and immediately inhibits mTOR, and any change in the AKT/mTOR pathway may contribute to the pathological alterations of HCC. Moreover, the PI3K/AKT/mTOR pathway has been shown to participate in mediating adipogenesis to protect cancer cells from oxidative stress and ferroptosis [27, 28]. MELK has also been shown to mediate the resuscitation of AKT/mTOR signaling to promote tumorigenesis in colorectal cancer [29]. In addition, Liu et al. [30] found that miR-21-5p also inhibited myeloma cell apoptosis by targeting RUNX1 and suppressing the resuscitation of the PI3K/AKT/mTOR pathway. Therefore, we hypothesized that miR-21-5p may inhibit the ferroptosis of HCC cells through the regulation of the AKT/mTOR signaling pathway by MELK.

In conclusion, we investigated the mechanism of miR-21-5p inhibiting the ferroptosis of HCC cells by regulating the AKT/mTOR pathway through MELK in this study, with the aim to provide a basis for clarifying the mechanism of hepatocarcinogenesis and progression, which may lead to new targets and ideas for the therapy of HCC.

## 2. Experimental Methods

### 2.1. Patient and Clinical Samples

Thirty pairs of HCC tissues and paracancerous tissues were obtained from the First People's Hospital of Kunming. All tissues were frozen in liquid nitrogen within 15 min after surgical resection and conserved at the specimen bank at −80°C. This study was approved by the ethics committee of Kunming First People's Hospital and conducted after informed consent was obtained from each subject.

### 2.2. Cell Treatment and Culture

Normal hepatocytes (QSG-7701) and hepatoma cells (HepG2, MHCC97H, MHCC97L, Huh-7, Hep3B) were purchased from Huatuo Biotech (Shenzhen, China). Cultures were incubated in Dulbecco's modified Eagle medium (DMEM) supplemented with 10% fetal bovine serum (FBS) and 1% streptomycin in an incubator at 37°C and 5% CO_2_. Microscopy was used to check the cell morphology, and the culture substrate was changed and passaged routinely. miR-21-5p mimic, mimic negative control (mimic NC), miR-21-5p suppressor, NC inhibitor NC transfected cells at ∼80% confluency. pCMV6-MELK-Myc-DDK (oe-MELK) and pCMV6-Myc-DDK (oe-NC) were purchased from ORIGENE and transfected into hepatoma cells for 24 hr to construct stable MELK-overexpressing hepatoma cell lines. G418 (1.2 mg/mL) was added to filter stable cell clones with high MELK expression, and the cells were cultured in DMEM substrate containing 10% FBS and 1.2 mg/mL G418.

### 2.3. Animal Modeling

Tumor cells in logarithmic growth phase (human hepatoma cell line HepG2) cultured under standard conditions were digested and centrifuged, and the concentration of the cell suspension was adjusted to 5 × 10^5^/mL with PBS. Fifty SPF athymic Balb/C nude mice (male, 4–6 weeks, 16–18 g) were purchased from the Animal Experimental Center of Kunming Medical University. The mice were injected with 3% pentobarbital sodium. After anesthetization, 100 *μ*L of the prepared cells was injected into the armpit of the mice. After waking up, the mice were fed a normal diet. The mice were sacrificed, and the growth of liver tumors was observed after 4 weeks.

### 2.4. RT-qPCR

Total RNA was separated from MHCC97L and HepG2 cells by TRIzol reagent, and the quality and concentration of RNA were measured using a NanoDrop 2,000. Then, a cDNA reverse transcriptase kit (Takara, Otsu, Japan) was used to reverse transcribe RNA into cDNA, followed by fluorescence quantitative PCR using the cDNA. The reaction program was set (95°C 10 min ⟶ 95°C 5 s ⟶ 63°C 30 s ⟶ 72°C 30 s ⟶ 72°C 5 min) × 40 as the amplification conditions. The internal reference genes were GAPDH and U6, and the relative level of the target product relative to the internal reference was expressed as 2^−*ΔΔ*Ct^ (three biological replicates and three technical replicates). The detailed primer sequences are in Table 1.

### 2.5. Western Blot

Total protein was separated from cells and tissues, and the total protein concentration was determined using the BCA method. Sample proteins were prepared, and the target proteins were separated with 10% SDS–PAGE. The separated proteins were transferred to PVDF membranes and blocked with 5% skim milk for 3 hr at room temperature, followed by the addition of primary antibodies against MELK (1 : 1,000), FTH1 (1 : 1,000), p-AKT (1 : 1,000), fibronectin (1 : 1,000), p-mTOR (1 : 2,000), N-cadherin (1 : 1,000), HO-1 (1 : 2,000), AKT (1 : 1,000), mTOR (1 : 2,000), vimentin (1 : 2,000), GPX4 (1 : 1,000), E-cadherin (1 : 2,000), and *x*CT (1 : 1,000). The next day, the corresponding secondary antibody was added and incubated at room temperature for 1 hr after rinsing the membrane. Chemiluminescent reagent was added to visualize the protein, and the gray value of the band was analyzed by ImageJ.

### 2.6. Fluorescence In Situ Hybridization (FISH)

The cells were fixed with 4% paraformaldehyde, washed with PBS, and then air-dried. The cells were immersed in 50%, 80%, and 90% ethanol solutions for 3 min and then air-dried at room temperature. Then, 10 *μ*L of probe were added to the sample, hybridized for 1.5 hr, rinsed with deionized water, and analyzed after drying. Fluorescence signal observation was performed using a fluorescence microscope. Fluorescence images of different colors were observed under the excitation of three-color fluorescence.

### 2.7. CCK-8

A CCK-8 kit was used to detect cell viability. Digested MHCC97L and HepG2 cells were digested into a cell suspension of 5 × 10^3^/mL and incubated in a 96-well plate (100 *μ*L/well). After adherent growth of the cells, each group of cells was treated with the corresponding transfection, and then the culture plates were precultured in an incubator (37°C, 5% CO_2_) for 0, 24, and 48 hr. Ten microliters of CCK-8 were added to each well, the culture plate was incubated in the incubator for 4 hr, and the absorbance value was measured at 450 nm with a microplate reader.

### 2.8. Clonogenic Assay

Each group of cells in logarithmic growth phase was digested with 0.25% trypsin and suspended in DMEM containing 10% FBS. Cells (1 × 10^5^/mL) were seeded in 24-well plates and cultured at 37°C for 2 weeks in a 5% CO_2_ incubator. The culture was stopped and the supernatant was discarded when visible clones were observed in the culture dish, and 4% paraformaldehyde was added to fix the cells. The cells were removed from fixation, dyed with crystal violet for 20 min, washed with running water to remove the staining solution, and dried in air. Photos were taken, and the cloning rate was calculated. Finally, the clone formation rate was computed, and the clone formation rate was equal to (the number of clones/inoculated cells) × 100%.

### 2.9. Transwell

#### 2.9.1. Migration Experiment

Cultured cells in logarithmic growth phase were digested with trypsin and suspended in serum-free substrate, and the cell concentration was adjusted to 2 × 10^5^/mL. Then, 600 *µ*L of substrate containing 10% serum was added to the lower chamber, 200 *µ*L of serum-free substrate was added to the upper chamber, and the cells were cultured in the incubator for 24 hr. The lower surface was immersed in 4% paraformaldehyde solution, fixed for 30 min, dyed with crystal violet, and observed and photographed with a microscope.

#### 2.9.2. Invasion Experiment

Cells in the logarithmic growth phase were digested with trypsin, suspended in serum-free medium, counted, and adjusted to a concentration of 2 × 10^5^/mL. Matrigel was diluted to 300 *μ*L/mL with serum-free cell culture medium at 4°C. Then, 100 *μ*L of Matrigel was applied evenly to the upper surface of the PET membrane of the cell culture insert, and the culture insert was gently placed into a 24-well plate, placed at 37°C for ∼3 hr, and then dried overnight. Then, 600 *μ*L of medium containing 10% serum was added to the lower chamber, 200 *μ*L of cell suspension was added to the upper chamber, and the cells were cultured in the incubator for 24 hr. The lower surface was immersed in 4% paraformaldehyde, fixed for 30 min, dyed with crystal violet, and observed using microscopy. The number of cells passing through the Matrigel glue on the lower surface of the PET film was calculated, the five visual fields in the middle and periphery were calculated, and the average value was taken.

### 2.10. EdU Staining

Staining was performed using the EdU Cell Proliferation Assay Kit (Thermo Fisher Scientific, USA). The same volume of EdU working solution and culture solution was added to a 96-well plate and incubated for 2 hr. Then, 50 *μ*L of cell fixative was added to each well and incubated at room temperature for 30 min. Then, 100 *μ*L of reaction solution was added to each well, kept away from light, and incubated at room temperature for 30 min in a decolorizing shaker. Images were observed and captured with a fluorescence inverted microscope.

### 2.11. Dual-Luciferase Reporter Gene

The PINK1 3′UTR fragment containing MELK 3′UTR-WT and MELK 3′UTR-MUT sequences was cloned into the pmir GLO vector using the dual-luciferase reporter gene detection kit. Forty-eight hours after transfection of GLO-MELK 3′UTR-WT and GLO-MELK 3′UTR-MUT, the luciferase activity was determined using a dual-luciferase reporter assay system.

### 2.12. Coimmunoprecipitation (Co-IP)

Total proteins were extracted from MHCC97L and HepG2 cells by total protein extraction buffer. Protein A/G Sepharose was preincubated with anti-MELK (1 : 1,000, CST) antibody on a shaker at 4°C for 60 min, followed by two washes. All IPs were incubated overnight at 4°C on a shaker. The precipitate was collected by centrifugation and then rinsed three times with lysis buffer. Immunoprecipitates were then subjected to immunoblot analysis.

### 2.13. ROS Detection

Flow cytometry was used to detect reactive oxygen species (ROS) content in MHCC97L and HepG2 cells. The cells in each group were plated after trypsin digestion into a six-well plate with the cell density adjusted by cell culture medium and then incubated in a cell incubator containing 5% CO_2_ for 24 hr. After centrifugation, the culture medium was discarded, and D-Hanks solution was added to resuspend the cells. D-Hanks solution incorporating DCFH-DA was added to each sample and incubated in a cell incubator for 30 min in darkness. The cells were centrifuged for 5 min and washed twice with D-Hanks solution. D-Hanks solution was added again to resuspend the cells, and the level of ROS in the cells was measured by flow cytometry.

### 2.14. Determination of Fe^2+^ Content

An iron detection kit was used to detect the Fe^2+^ content in the cells according to the protocol provided by the manufacturer.

### 2.15. Hematoxylin and Eosin Staining

Tissue samples were dewaxed with xylene, rinsed with 100%, 90%, 80%, and 70% alcohol for 5 min, and rinsed with PBS. Then, hematoxylin and eosin staining was performed for 5 min and rinsed with running water. The cells were differentiated with 5% acetic acid for 1 min, rinsed with running water, treated with bluing solution for 1 min, stained with eosin for 1 min, and rinsed with running water. Then, the sections were cleared with 70%, 80%, 90%, and 100% alcohol, soaked in xylene for transparency, dried at room temperature, sealed with neutral gum, and observed and imaged under a microscope.

### 2.16. Bioinformatics Analysis

The differentially expressed genes were obtained from the bioinformatics database GEO datasets (https://www.ncbi.nlm.nih.gov/) and then analyzed using edgeR, and a miRNA differential heatmap and volcano map were drawn. Targeted binding sites between miR-21-5p and MELK were determined using starBase.

### 2.17. Statistical Analysis

GraphPad Prism 8.0 was used to analyze the experimental data and plot the graphs. The analysis results are expressed as the mean ± SD. Each experiment was performed with at least three replicates. One-way analysis of variance and *t*-test were used for means analysis. *P* < 0.05 and *P* < 0.01 were considered statistically significant.

## 3. Results

### 3.1. miR-21-5p and MELK Are Highly Expressed in HCC

**Figure 1:**
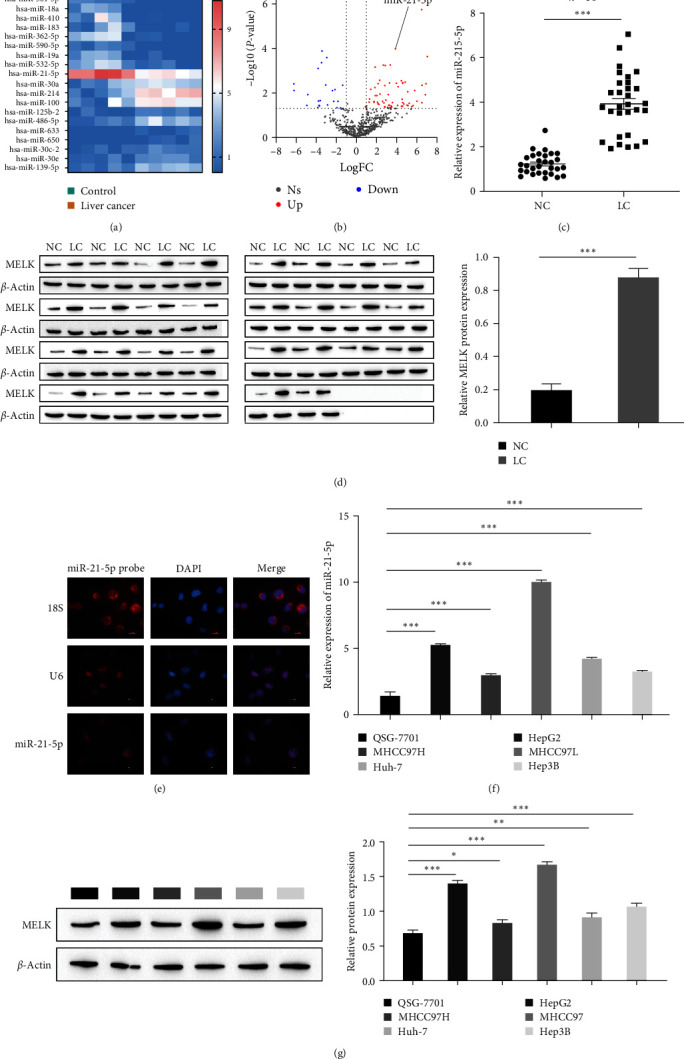
High levels of miR-21-5p and MELK in hepatocellular carcinoma: (a) differential heatmap of miRNA expression in liver cancer; (b) volcano plot of miRNA expression in liver cancer; (c) RT-qPCR was used to detect miR-21-5p expression in pathological samples; (d) Western blot was used to detect MELK expression in pathological samples; (e) FISH for the level of miR-21-5p in liver cancer cells; (f) RT-qPCR for the level of miR-21-5p (normal liver cells QSG-7701, hepatoma cells HepG2, MHCC97H, MHCC97L, Huh-7, Hep3B); (g) Western blot for the level of MELK (normal liver cells QSG-7701, liver cancer cells HepG2, MHCC97H, MHCC97L, Huh-7, Hep3B).  ^*∗*^*P* < 0.05,  ^*∗∗*^*P* < 0.01,  ^*∗∗∗*^*P* < 0.001.

First, the bioinformatics database GEO datasets (https://www.ncbi.nlm.nih.gov/) revealed that the expression of miR-21-5p was significantly upregulated in HCC (Figures 1(a) and 1(b)). The RT-qPCR and Western blot results of the levels of miR-21-5p and MELK in 30 groups of clinicopathological samples (HCC tissues and paracancerous tissues of HCC patients) showed that the levels of miR-21-5p and MELK were markedly elevated in HCC (Figures 1(c) and 1(d)). The FISH results showed that miR-21-5p was expressed in isolated hepatoma cells and mainly distributed in the nucleus (Figure 1(e)). Next, we examined the level of miR-21-5p in normal hepatocytes (QSG-7701) and five hepatoma cell lines (HepG2, MHCC97H, MHCC97L, Huh-7, Hep3B) by RT-qPCR, and the results showed that compared with QSG-7701 cells, miR-21-5p was expressed at a significantly higher level in five HCC cell lines, especially in MHCC97L and HepG2 cells (Figure 1(f)). Western blot results showed that the level of MELK was higher in MHCC97L and HepG2 cells (Figure 1(g)). Therefore, we selected MHCC97L and HepG2 cells for further study.

### 3.2. Overexpression of miR-21-5p and MELK Can Facilitate EMT in Hepatocellular Carcinoma Cells

**Figure 2:**
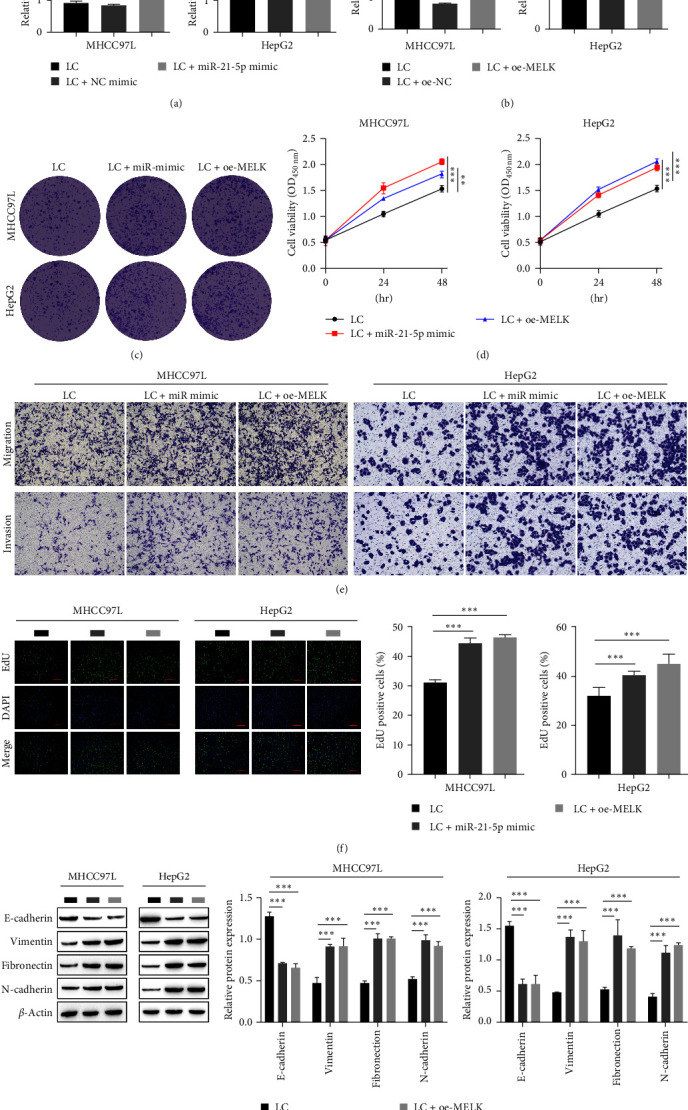
Overexpression of miR-21-5p and MELK can facilitate EMT in hepatocellular carcinoma cells: (a) transfection efficiency of miR-21-5p mimic by RT-qPCR; (b) RT-qPCR for detecting transfection efficiency of oe-MELK; (c) clone formation assay for cell proliferation; (d) cell viability by CCK8; (e) transwell assay for cell invasion and migration; (f) EdU staining for cell proliferation; (g) Western blot for the level of EMT-related proteins.  ^*∗∗*^*P* < 0.01,  ^*∗∗∗*^*P* < 0.001.

To further investigate the influence of miR-21-5p and MELK overexpression on HCC cells, we transfected MHCC97L and HepG2 cells with miR-21-5p mimic and oe-MELK. The transfection efficiency of the miR-21-5p mimic and oe-MELK was detected by RT-qPCR (Figures 2(a) and 2(b)), indicating that the transfection was successful. Next, clonogenic experiments showed a prominent elevation in the clonogenic rate of MHCC97L and HepG2 cells after miR-21-5p and MELK overexpression (Figure 2(c)). The CCK-8 results showed that overexpression of miR-21-5p and MELK observably enhanced the cell viability of HCC cells compared with normal hepatocytes (Figure 2(d)), indicating that miR-21-5p and MELK promoted the proliferation of HCC cells. The transwell assay results showed that the overexpression of miR-21-5p and MELK promoted the invasion and migration of HCC cells, significantly exacerbating cell migration (Figure 2(e)). Using an EdU assay, we also observed that overexpression of miR-21-5p and MELK elevated the percentage of EdU-positive cells in MHCC97L and HepG2 cells (Figure 2(f)), indicating that HCC cells had significantly enhanced proliferative capacity. The Western blot results showed that overexpression of miR-21-5p and MELK increased the levels of vimentin, fibronectin, N-cadherin, and decreased the level of E-cadherin (Figure 2(g)), illustrating that overexpression of miR-21-5p and MELK promoted EMT in HCC cells.

### 3.3. miR-21-5p Targets MELK, and Downregulation of miR-21-5p Can Inhibit the Expression of MELK and the Progression of HCC

**Figure 3:**
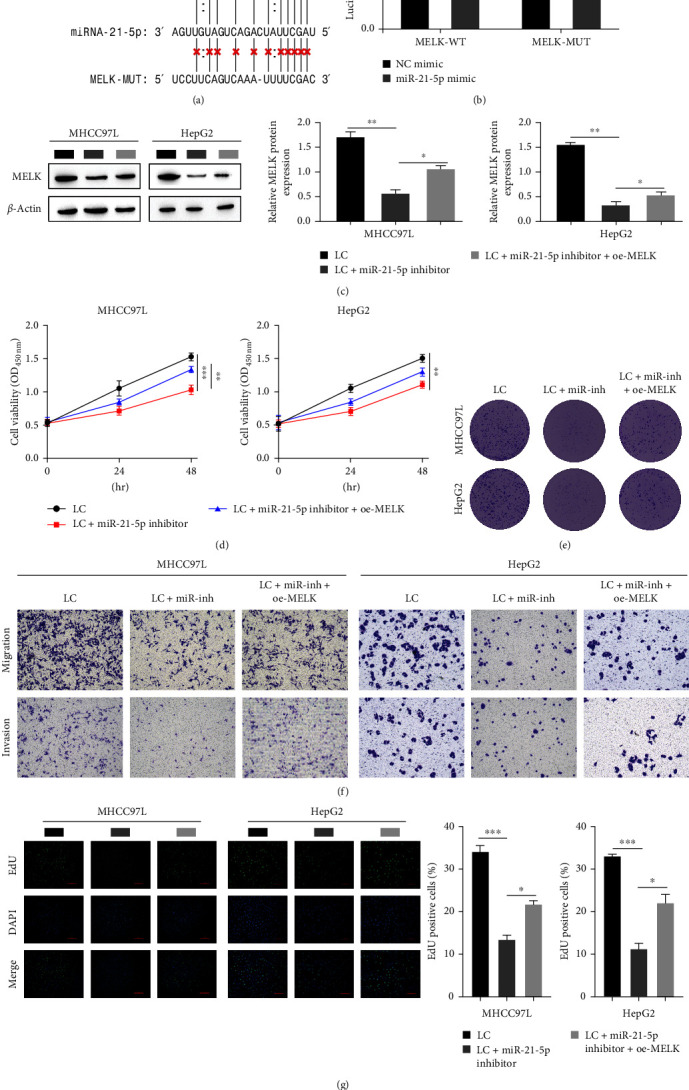
miR-21-5p targets MELK, and miR-21-5p can suppress the expression of MELK, thereby inhibiting tumor progression: (a) targeted binding sites of miR-21-5p and MELK predicted by starBase; (b) dual-luciferase reporter gene experiment verifying the targeted relationship between miR-21-5p and MELK; (c) Western blot for the level of MELK; (d) CCK8 for cell viability; (e) clonogenic assay for cell proliferation; (f) transwell assay for invasion and migration; (g) EdU staining for cell proliferation.  ^*∗*^*P* < 0.05,  ^*∗∗*^*P* < 0.01,  ^*∗∗∗*^*P* < 0.001.

It is well known that miRNAs are involved in posttranscriptional gene expression regulation by matching with complementary sequences of the 3′UTR on target mRNA transcripts. Therefore, we examined the molecular mechanism of miR-21-5p and the miRNA‒mRNA interaction. First, a targeted binding site between miR-21-5p and MELK was identified by StarBase (Figure 3(a)). Next, the dual-luciferase reporter results showed that the luciferase activity of MELK 3′ UTR-WT was observably attenuated by miR-21-5p compared with the control group, while the luciferase activity of MELK 3′UTR-WUT was not dramatically altered (Figure 3(b)), which demonstrates that miR-21-5p targeted MELK binding. To ultimately demonstrate the influence of MELK regulation by miR-21-5p on HCC, we transfected MHCC97L and HepG2 cells with miR-21-5p to inhibit and overexpress MELK. The level of MELK was significantly reduced after transfection with a miR-21-5p inhibitor, as shown by Western blot, and overexpression of MELK after transfection with a miR-21-5p inhibitor increased the level of MELK (Figure 3(c)). The CCK-8 results showed that inhibition of miR-21-5p led to an observable decrease in HCC cell viability, while the overexpression of MELK after miR-21-5p inhibition led to an observable increase in cell viability (Figure 3(d)). Clonogenic assays showed that downregulation of miR-21-5p decreased the clonogenic efficiency of HCC cells, while downregulation of miR-21-5p observably elevated the clonogenic efficiency of MELK-overexpressing cells (Figure 3(e)). The transwell results showed that the invasion and migration of cells were observably reduced after inhibition of miR-21-5p, and overexpression of MELK after inhibition of miR-21-5p increased invasion and migration (Figure 3(f)). The results of the EdU assay showed that the inhibition of miR-21-5p decreased the proliferative capacity of cells, and the overexpression of MELK after the inhibition of miR-21-5p led to an observable increase in the proliferation ability of cells (Figure 3(g)). These results suggest that downregulation of miR-21-5p suppresses the level of MELK and inhibits the proliferation, invasion and migration of HCC cells, thereby inhibiting the progression of HCC and promoting the ferroptosis of HCC cells.

### 3.4. MELK Inhibits Ferroptosis in Hepatocellular Carcinoma Cells via AKT/mTOR

**Figure 4:**
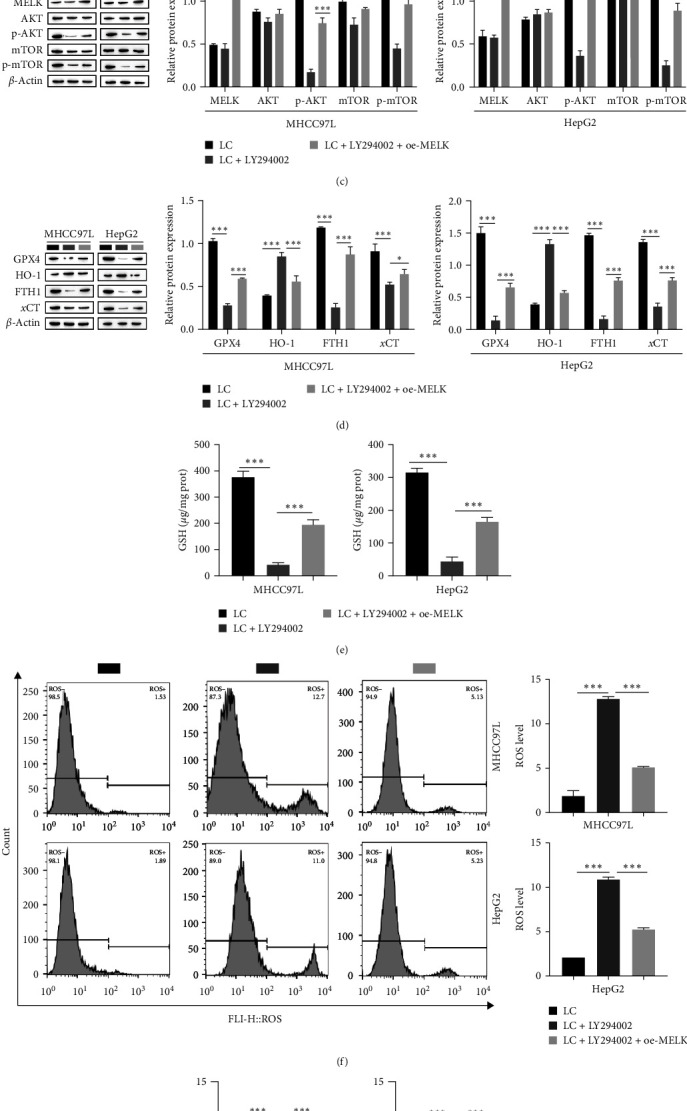
MELK inhibits liver cancer cell ferroptosis through AKT/mTOR: (a) coimmunoprecipitation to verify the interaction between MELK and AKT; (b) CCK8 for cell viability; (c) Western blot for the level of MELK, AKT, mTOR, p-AKT, and p-mTOR; (d) Western blot for the level of ferroptosis-related proteins GPX4, HO-1, FTH1, and *x*CT; (e) kit assay for the level of GSH; (f) flow cytometry to detect ROS content; (g) kit assay to detect the Fe^2+^ content,  ^*∗*^*P* < 0.05,  ^*∗∗*^*P* < 0.01,  ^*∗∗∗*^*P* < 0.001.

To further investigate the molecular mechanism of ferroptosis in HCC cells, we used MELK antibody for coimmunoprecipitation, and Western blot analysis of the precipitate showed that AKT was present in the precipitate (Figure 4(a)), demonstrating the interaction between MELK and AKT. Next, to investigate the influence of MELK on the regulation of AKT on ferroptosis in HCC cells, we overexpressed MELK in MHCC97L and HepG2 cells and added the AKT inhibitor LY294002. The CCK-8 results showed that the addition of LY294002 led to a noticeable decrease in HCC cell viability, while overexpression of MELK reversed the effect of LY294002 and the cell viability increased significantly (Figure 4(b)). Western blot analysis showed that the levels of p-AKT and p-mTOR were observably decreased after LY294002 was added, while the levels of MELK, AKT, and mTOR were not markedly changed. However, the levels of MELK, p-AKT, and p-mTOR in MELK-overexpressing cells were markedly elevated after LY294002 was added, and there was no significant change in AKT or mTOR (Figure 4(c)). The Western blot results of GPX4, HO-1, FTH1, and *x*CT showed that the levels of GPX4, FTH1, and *x*CT were significantly decreased, and the level of HO-1 was significantly elevated by LY294002. Overexpression of MELK after the addition of LY294002 resulted in elevated levels of GPX4, FTH1, and *x*CT, and decreased expression of HO-1 (Figure 4(d)). The level of GSH was markedly decreased upon the addition of LY294002, as detected by the GSH kit, and overexpression of MELK upon the addition of LY294002 in turn increased the level of GSH (Figure 4(e)). The results of the flow cytometry analysis showed that the intracellular ROS level was significantly increased after LY294002 addition, while the intracellular ROS level of MELK-overexpressing cells was decreased after LY294002 addition (Figure 4(f)). Fe^2+^ within HCC cells was significantly increased upon the addition of LY294002, as detected by the Fe^2+^ assay kit, which was, in turn, decreased in cells overexpressing MELK upon the addition of LY294002 (Figure 4(g)). These results suggest that MELK inhibits ferroptosis in HCC cells by rescuing the AKT/mTOR pathway.

### 3.5. Erastin Weakens the Effect of miR-21-5p on the Ferroptosis of Hepatoma Cells and the Level of EMT

**Figure 5:**
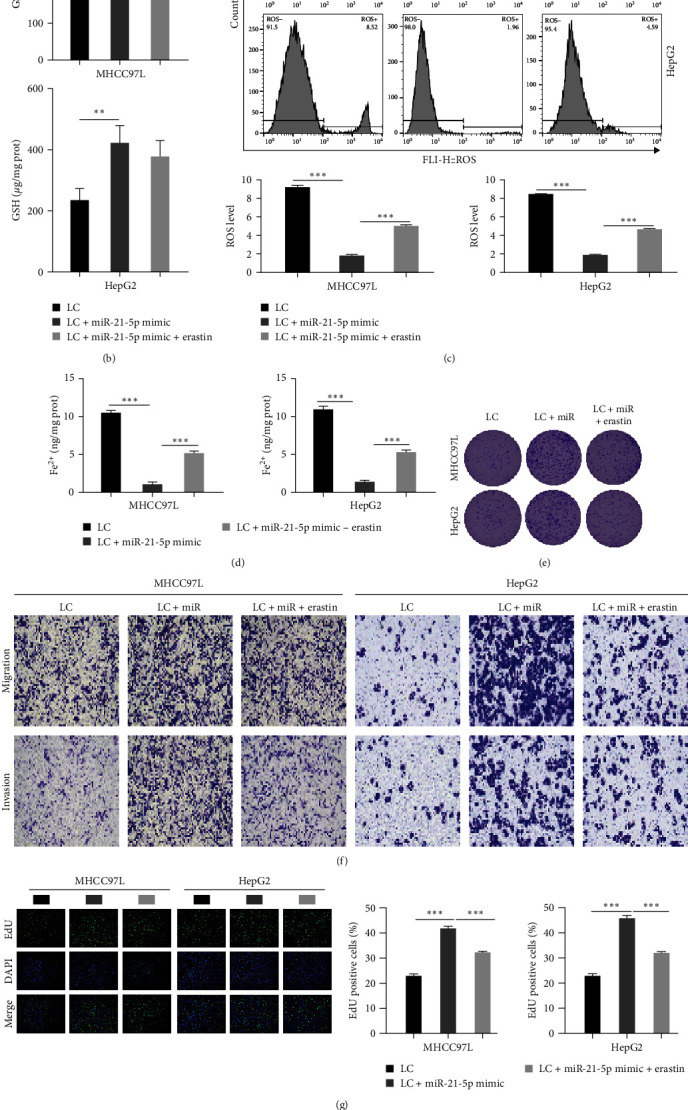
After the addition of an ferroptosis inducer, the effect of miR-21-5p on the ferroptosis of hepatoma cells and the level of EMT was weakened: (a) Western blot analysis of the level of GPX4, HO-1, FTH1, *x*CT; (b) kit assay for detecting the level of GSH; (c) flow cytometry for ROS content; (d) kit assay to detect Fe^2+^content; (e) clone formation assay for detecting cell proliferation; (f) transwell assay for detecting invasion and migration; (g) EdU staining to detect cell proliferation; (h) Western blot assay of the expression of E-cadherin, vimentin, fibronection, and N-cadherin,  ^*∗*^*P* < 0.05,  ^*∗∗*^*P* < 0.01,  ^*∗∗∗*^*P* < 0.001.

To verify the influence of miR-21-5p on ferroptosis and EMT in HCC cells, we transfected MHCC97L and HepG2 cells with miR-21-5p mimic and added erastin, an ferroptosis inducer. The Western blot results showed that the levels of GPX4, FTH1, and *x*CT were markedly elevated after transfection of the miR-21-5p mimic, while the level of HO-1 was significantly decreased. The addition of the ferroptosis inducer erastin significantly inhibited the overexpression of miR-21-5p, decreased the levels of GPX4, FTH1, and *x*CT, and increased the expression of HO-1 (Figure 5(a)). The content of GSH was elevated after transfection of the miR-21-5p mimic and decreased after the addition of erastin, as detected using the GSH kit (Figure 5(b)). In addition, the flow cytometry results showed that transfection of the miR-21-5p mimic induced a noticeable reduction in ROS levels, and the addition of erastin induced an increase in ROS levels (Figure 5(c)). The results of the Fe^2+^ content assay in HCC cells demonstrated a significant decrease after transfection of the miR-21-5p mimic and then a significant increase after the addition of the ferroptosis inducer erastin (Figure 5(d)). This suggests that the ferroptosis inducer erastin attenuates the repressive influence of miR-21-5p on ferroptosis in HCC cells. Next, the clonogenic assay showed that the clonogenic rate of HCC cells increased markedly after miR-21-5p overexpression and decreased significantly after erastin was added (Figure 5(e)). The transwell assay showed that miR-21-5p overexpression facilitated the invasion and migration of HCC cells, and the invasion and migration ability decreased significantly after adding erastin (Figure 5(f)). The EdU assay also confirmed that overexpression of miR-21-5p increased the percentage of EdU-positive cells in HCC cells, whereas the addition of erastin induced a remarkable reduction in the percentage of EdU-positive cells (Figure 5(g)), indicating that miR-21-5p facilitated HCC cell proliferation, whereas the addition of erastin attenuated this enhancement. Moreover, the level of E-cadherin decreased, and the levels of vimentin, fibronectin, and N-cadherin increased after miR-21-5p overexpression, as shown by Western blotting. The addition of erastin reversed this effect (Figure 5(h)), indicating that overexpression of miR-21-5p promoted EMT in HCC cells, and erastin, an ferroptosis inducer, attenuated the promotion of EMT by miR-21-5p in HCC cells.

### 3.6. miR-21-5p Inhibits Ferroptosis in Hepatocellular Carcinoma Cells by Regulating the AKT/mTOR Signaling Pathway through MELK In Vivo

**Figure 6:**
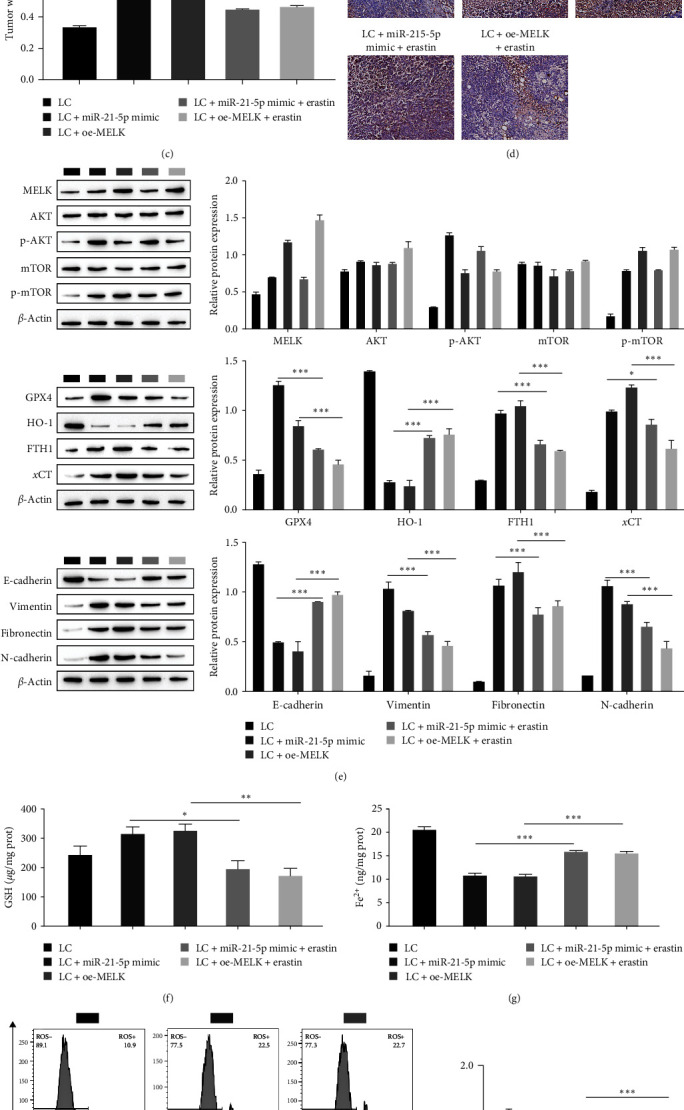
Animal experiments demonstrate that miR-21-5p inhibits ferroptosis in hepatocellular carcinoma cells by regulating the AKT/mTOR pathway through MELK: (a) tumor size in nude mice; (b) tumor growth curve; (c) tumor weight in nude mice; (d) staining of immunohistochemical Ki-67; (e) pathway-related proteins MELK, AKT, mTOR, p-AKT, p-mTOR, and ferroptosis-related proteins GPX4, HO-1, FTH1, and *x*CT were detected by Western blot. The levels of E-cadherin, vimentin, fibronectin, and N-cadherin; (f) kit assay of the level of GSH; (g) kit assay of Fe^2+^ content; (h) kit assay of ROS content,  ^*∗*^*P* < 0.05,  ^*∗∗*^*P* < 0.01,  ^*∗∗∗*^*P* < 0.001.

Using a subcutaneous tumor formation model in nude mice, we observed that miR-21-5p inhibits the ferroptosis of hepatoma cells by regulating the AKT/mTOR signaling pathway through MELK in vivo. HCC tumors were enlarged in the LC + miR-21-5p mimic and LC + OE-MELK groups compared to the LC group, as observed by tumor size in nude mice, and progression of HCC was significantly reduced after addition of the ferroptosis inducer erastin (Figure 6(a)). The tumor growth curve showed that compared with the LC group, the tumors in the LC + miR-21-5p mimic and LC + OE-MELK groups grew rapidly, while tumor growth was significantly slowed after the addition of erastin (Figure 6(b)). The tumor weight measurement results showed that the tumor weight was observably elevated in the LC + miR-21-5p mimic and LC + OE-MELK groups compared with the LC group, while the tumor weight was decreased following the addition of erastin (Figure 6(c)). Immunohistochemical Ki-67 results showed that tumor proliferation was dramatically enhanced in the LC + miR-21-5p mimic and LC + OE-MELK groups compared with the LC group. Tumor proliferation in the LC + miR-21-5p mimic + erastin and LC + OE-MELK + erastin groups was observably reduced compared with that in the LC + miR-21-5p mimic and LC + OE-MELK groups (Figure 6(d)). In addition, Western blot analysis showed that the levels of MELK, p-AKT, and p-mTOR were observably elevated after overexpression of miR-21-5p and MELK, while AKT and mTOR had no significant change. There were no marked alterations in MELK, AKT, mTOR, p-AKT, and p-mTOR after the addition of erastin, demonstrating that miR-21-5p regulated the AKT/mTOR pathway through MELK, while the activity of the AKT/mTOR signaling pathway was not affected by erastin. In addition, Western blot results showed that the levels of the ferroptosis-related proteins GPX4, FTH1, and *x*CT increased significantly after miR-21-5p and MELK overexpression, HO-1 expression decreased significantly, and the levels of GPX4, FTH1, and *x*CT decreased significantly after erastin was added. The level of HO-1 was observably increased. After the overexpression of miR-21-5p and MELK, the content of E-cadherin decreased, while the content of vimentin, fibronectin, and N-cadherin increased, and the level of E-cadherin was enhanced by adding erastin. The expression of vimentin, fibronectin, and N-cadherin was downregulated (Figure 6(e)). Next, the GSH and ROS assay results showed that miR-21-5p and MELK overexpression facilitated GSH expression and inhibited ROS and Fe^2+^ levels, while the addition of erastin inhibited GSH expression and increased ROS and Fe^2+^ levels (Figure 6(f)–6(h)).

## 4. Discussion

HCC is a common and highly refractory cancer. Although rapid advances in diagnosis and therapy have perfected the prognosis of patients, the survival benefit is mainly seen in patients with early stage and intermediate-stage HCC, and the treatment of advanced HCC remains challenging [31, 32]. At present, the standard first-line drug for advanced liver cancer is sorafenib, but due to its drug resistance and other problems, the impact on overall survival and tumor progression is limited [33, 34]. Therefore, it is imperative to find novel and effective remedial strategies for the treatment of HCC, and exploring the molecular mechanism of the occurrence and progression of HCC may provide a reference for finding new therapeutic strategies. For the past few years, a number of studies have identified the critical role of ferroptosis in inhibiting tumor growth [35, 36]. Earlier studies have also found that ferroptosis is important for the treatment and prognosis of liver cancer [5]. However, the key signaling pathways and regulators of ferroptosis in the evolution and progression of HCC are still unclear. In this study, we demonstrate that miR-21-5p inhibits ferroptosis in HCC cells through the regulation of AKT/mTOR signaling by MELK. This discovery will contribute to a better understanding of the regulatory mechanism of ferroptosis in HCC cells.

miRNAs are often singularly expressed in cancer cells and impact the regulation of gene expression at the posttranscriptional level by matching with complementary sequences of the 3′UTR on target mRNA transcripts and participate in the occurrence and evolution of tumors as cancer activators or suppressors [37]. For example, miR-15a-3p inhibits metastasis of HCC by interacting with HMOX1 [38]. miR-22-3p promotes HCC progression by targeting CDKN2C and contributes to poor HCC prognosis [39]. Exosomal miR-21 can regulate the expression of the tumor suppressor genes PTEN and PTENp1 and affect the growth of HCC cells [40], and miR-21-3p promotes HCC progression via SMAD7/YAP1 regulation [41]. Using bioinformatics analysis and clinicopathological analysis, we found that miR-21-5p and MELK were highly expressed in HCC. We then overexpressed miR-21-5p and MELK in two HCC cell lines (MHCC97L and HepG2) and found that the proliferation, colony formation, EMT, invasion, and migration capacities of the two cell lines were enhanced. Next, the starBase prediction results showed that there was a targeted binding site between miR-21-5p and MELK, and we verified the binding effect between them by dual-luciferase gene analysis. In addition, we observed that the expression of MELK was suppressed after miR-21-5p was downregulated in two HCC cell lines, indicating that miR-21-5p positively regulates the level of MELK. Thus, the superabundantly expressed miR-21-5p facilitates tumor progression through MELK in HCC.

Ferroptosis, characterized by excess iron accumulation and lipid peroxidation, is a novel modality of iron-dependent cell death. Increasing evidence supports a critical role of ferroptosis in the inhibition of liver cancer [42, 43]. It has been reported that miR-23a-3p directly targets ACSL4 to regulate ferroptosis to improve sorafenib resistance and inhibit liver cancer [44]. miR-362-3p can promote the expression of MIOX and increase ferroptosis to alleviate the progression of liver cancer [45]. miR-3200-5p regulates liver cancer progression by targeting ATF4 to induce ferroptosis [46]. In recent years, several mediators involved in ferroptosis have been reported, including the accumulation of ROS, consumption of GSH, suppression of GPX4 activity, and elevated release of polyunsaturated fatty acids [47–49]. In addition, HO-1 [47], iron storage protein 1 (FHT1) [50], and *x*CT [51] also mediate the occurrence of ferroptosis. In this study, we observed that the levels of MELK, GPX4, GSH, FTH1, and *x*CT decreased, the levels of HO-1, ROS, and Fe^2+^ increased after miR-21-5p was downregulated in HCC cells. Therefore, miR-21-5p can facilitate the ferroptosis of liver cancer cells by inhibiting the level of MELK, thereby inhibiting the progression of liver cancer.

In a previous study, Yao et al. [52] found that targeting the LIFR/NF-*κ*B/LCN2 axis controlled liver tumorigenesis and susceptibility to ferroptosis. Yi et al. [27] reported that suppression of the PI3K-AKT-mTOR pathway can induce ferroptosis in HCC cells. Cheng et al. [53] showed that semaphorin 5A can regulate the AKT-mTOR signaling pathway and inhibit ferroptosis. Li et al. [54] found that melatonin could also inhibit ferroptosis by regulating the AKT-mTOR signaling pathway. We found a targeted interaction between MELK and AKT/mTOR signaling by coimmunoprecipitation. After using the AKT inhibitor LY294002, we found that LY294002 inhibited the levels of GPX4, GSH, FTH1, and *x*CT, and promoted an increase in HO-1, ROS, and Fe^2+^ content in HCC cells, but these results were reversed after overexpressing MELK. The results showed that MELK inhibited the ferroptosis of HCC cells by rescuing the AKT/mTOR pathway.

Erastin is an inducer of ferroptosis that can trigger multiple regulatory molecules with high efficiency and fast and lasting effects [55]. Erastin has been found to enhance sensitivity to chemotherapy and radiotherapy and has the potential to be used as an anticancer drug [56]. In this study, erastin was found to reverse the inhibitory influence of miR-21-5p on ferroptosis in HCC cells. In addition, in vivo, we observed that overexpression of both miR-21-5p and MELK resulted in tumor enlargement, rapid growth, and increased weight in nude mice, which was significantly alleviated by the addition of erastin. We also found that erastin was able to reverse the influence of miR-21-5p in terms of EMT levels in mouse tumors, changes in ferroptosis-related mediators, and signaling pathway activity. Therefore, our results demonstrate that miR-21-5p promotes HCC progression by inhibiting ferroptosis in HCC cells through MELK regulation of the AKT/mTOR signaling pathway.

In short, this study elucidated the key mechanism by which miR-21-5p inhibits ferroptosis in HCC cells through regulation of the AKT/mTOR pathway by MELK at the cellular and organismal levels. Overexpression of miR-21-5p upregulates the level of MELK to activate the AKT/mTOR pathway, thereby inhibiting the ferroptosis process of HCC cells and promoting the growth of HCC. Although a growing number of studies have focused on investigating the molecular mechanisms and signaling pathways of ferroptosis during HCC, there are still many questions that need to be addressed, such as the exact molecular events involved in the final cell death induced by ferroptosis that have not yet been fully elucidated. Improving the understanding of ferroptosis is important for comprehending the physiological function and remedial latency of this cell death mode. This study provides a basis for the clinical diagnosis and therapy of HCC, but further research is still needed at the clinical and mechanistic levels.

## Figures and Tables

**Table 1:** RT-qPCR primer sequence.

Genes	Forward primer (5′-3′)	Reverse primer (5′-3′)
miR-21-5p	AGUUGUAGUCAGACUAUUCGAU	CUUUACGAUGAAUUCAUUUCC
MELK	GCT GCA AGG TAT AAT TGA TGGA	CAG TAA CAT AAT GAC AGA TGGGC
U6	CGATACAGAGAAGATTACATGGC	AACGCTTCACGAATTTGCGT
GAPDH	ACAGTCAGCCGCATCTTCTT	GTTAAAAGCAGCCCTGGTA

## Data Availability

The data used to support the findings of this study are included within the article.

## Abbreviations

AKT: Protein kinase B
mTOR: Mammalian target of rapamycin
HCC: Hepatocellular carcinoma
EdU: 5-ethynyl-2′-deoxyuridine
MELK: Maternal–fetal leucine zipper kinase
PI3K: Phosphatidylinositol-3-kinase
ROS: Reactive oxygen species
GPX4: Glutathione peroxidase 4
GSH: Glutathione SH
FTH1: Ferritin heavy chain 1
*x*CT: Cystine/glutamate antiporter solute carrier family 7 member 11.
